# A DNA Vaccine That Targets Hemagglutinin to Antigen-Presenting Cells Protects Mice against H7 Influenza

**DOI:** 10.1128/JVI.01340-17

**Published:** 2017-11-14

**Authors:** Tor Kristian Andersen, Fan Zhou, Rebecca Cox, Bjarne Bogen, Gunnveig Grødeland

**Affiliations:** a K. G. Jebsen Centre for Influenza Vaccine Research, University of Oslo and Oslo University Hospital, Oslo, Norway; b Institute of Clinical Medicine, University of Oslo and Oslo University Hospital, Oslo, Norway; c Influenza Centre, Department of Clinical Science, University of Bergen, Bergen, Norway; d Department of Research and Development, Haukeland University Hospital, Bergen, Norway; e Centre for Immune Regulation, University of Oslo, Oslo, Norway; Hudson Institute of Medical Research

**Keywords:** APC-targeting, DNA vaccine, avian viruses, hemagglutinin, influenza, pandemic influenza

## Abstract

Zoonotic influenza H7 viral infections have a case fatality rate of about 40%. Currently, no or limited human to human spread has occurred, but we may be facing a severe pandemic threat if the virus acquires the ability to transmit between humans. Novel vaccines that can be rapidly produced for global distribution are urgently needed, and DNA vaccines may be the only type of vaccine that allows for the speed necessary to quench an emerging pandemic. Here, we constructed DNA vaccines encoding the hemagglutinin (HA) from influenza A/chicken/Italy/13474/99 (H7N1). In order to increase the efficacy of DNA vaccination, HA was targeted to either major histocompatibility complex class II molecules or chemokine receptors 1, 3, and 5 (CCR1/3/5) that are expressed on antigen-presenting cells (APC). A single DNA vaccination with APC-targeted HA significantly increased antibody levels in sera compared to nontargeted control vaccines. The antibodies were confirmed neutralizing in an H7 pseudotype-based neutralization assay. Furthermore, the APC-targeted vaccines increased the levels of antigen-specific cytotoxic T cells, and a single DNA vaccination could confer protection against a lethal challenge with influenza A/turkey/Italy/3889/1999 (H7N1) in mice. In conclusion, we have developed a vaccine that rapidly could contribute protection against a pandemic threat from avian influenza.

**IMPORTANCE** Highly pathogenic avian influenza H7 constitute a pandemic threat that can cause severe illness and death in infected individuals. Vaccination is the main method of prophylaxis against influenza, but current vaccine strategies fall short in a pandemic situation due to a prolonged production time and insufficient production capabilities. In contrast, a DNA vaccine can be rapidly produced and deployed to prevent the potential escalation of a highly pathogenic influenza pandemic. We here demonstrate that a single DNA delivery of hemagglutinin from an H7 influenza could mediate full protection against a lethal challenge with H7N1 influenza in mice. Vaccine efficacy was contingent on targeting of the secreted vaccine protein to antigen-presenting cells.

## INTRODUCTION

Highly pathogenic avian influenza viruses (HPAIV) represent a potential pandemic threat. As of June, 2017, the World Health Organization has reported a total of 1,533 laboratory-confirmed cases of human infection with avian influenza H7N9, with a mortality rate of nearly 40% (1). A majority of these cases arose from zoonotic transmissions at the human-animal interface, with limited human-to-human transmission. Viruses isolated from human cases, perhaps including secondary cases of human transmissions, show only a few accumulated mutations in surface glycoproteins (2). Thus, it is difficult to predict the antigenic determinants of transmissibility that are needed to break the zoonotic barrier and also how these would influence the viral pathogenicity in humans (3). However, the human population is presently serologically naive toward H7 influenza; therefore, the potential acquisition of mutations enabling efficient human-to-human transmission could have a devastating effect.

Conventional vaccine design relies on an extensive surveillance system to determine which influenza strains will be included in the next season's influenza vaccine. The 2009 pandemic demonstrated that the development and vaccine production process could be completed in about 6 months, which represents a best-case scenario (4). Both the 2009 H1N1 pandemic and the 2013 HPAIV H7N9 emergence in China (5) demonstrate that it is difficult to predict which influenza strain will cause the next pandemic and that conventional influenza vaccines are not sufficient in the face of a pandemic outbreak. Novel vaccine formats that can rapidly be produced and quickly induce an immune response upon a novel pandemic threat are urgently needed (6–8).

The multifaceted pathogenicity of HPAIV is maintained by two major determinants. First, the hemagglutinin (HA) in HAPIV has a receptor binding preference for α2,3-linked sialic acid that is abundant on the gut epithelia of aquatic birds (9). In humans, α2,3- and α2,6-linked sialic acid receptors dominate in the lower and upper respiratory tract, respectively. Efficient human-to-human transmission of influenza virus is dependent on viral replication in the upper respiratory tract (10). The viral preference for α2,3-linked sialic acid receptors thus represents a natural barrier for zoonotic and human-to-human transmission with HPAIV (11). However, certain H7 isolates have been demonstrated to bind both sialic acid receptors (12–14), forming a breach in the zoonotic barrier (15). Second, HA in HPAIV have acquired a multibasic cleavage site (MBCS) (16–19). HA cleavage is necessary for influenza infectivity, and where seasonal influenza HAs are only cleaved by tissue-restricted proteases, HAPIV HAs can be cleaved by ubiquitous cellular proteases. Thus, the potential pathogenicity of an HPAIV is enhanced since the virus evades tissue-restricted replication (20–23).

DNA vaccines can be rapidly produced but are typically hampered by reduced immunogenicity. Previously, we demonstrated that a single DNA vaccination with HA from influenza H1N1 targeted to major histocompatibility complex class II (MHC-II) molecules on antigen-presenting cells (APCs) confers sterilizing immunity against influenza challenge in mice (24, 25). Furthermore, translation into larger animals confirmed the increased immunogenicity after APC-targeting of antigen (26). In addition to MHC-II targeting, chemokines are attractive targeting units due to the chemotactic recruitment of, among others, dendritic cells (DCs), macrophages, and NK cells, and chemokines can channel recruitment of internalized antigen to MHC-I and MHC-II presentation pathways (27–31). Here, we extended these experiments to vaccination against HPAIV H7. After targeting of HA from A/chicken/Italy/13474/1999 (H7N1) or A/turkey/Italy/3889/1999 (H7N1) to MHC-II molecules or chemokine receptors 1, 3, and 5 (CCR1/3/5) expressed on APC, we demonstrate that a single DNA vaccination can confer protection against a lethal H7N1 challenge in mice.

## RESULTS

### Construction and characterization of APC-targeted influenza vaccines.

**FIG 1:**
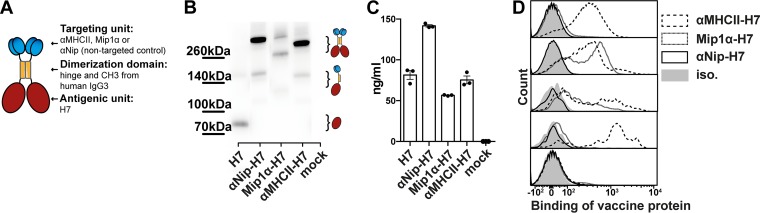
Characterization of vaccine proteins. (A) Schematic illustration of a dimeric vaccine protein. The targeting units, either an scFv specific for mouse I-E^d^ (αMHCII), the chemokine MIP1α (Mip1α), or a scFv specific for the hapten NIP (αNip; nontargeted control), are connected to HA via a dimerization unit containing the hinge and C_H_3 domain from human IgG3. (B) Western blot of supernatants from 293E cells transfected with the indicated vaccine plasmids. Molecular sizes and corresponding structures are indicated. (C) Vaccine proteins in supernatants from transiently transfected 293E cells were detected in an ELISA specific for HA from A/Shanghai/1/2013 (H7N9). (D) Binding of vaccine proteins to B cells (CD19^+^), macrophages (F4/80^+^ CD64^+^), DCs (Lin^−^ CD11c^hi^) divided into conventional DC1 (CD24^+^) and conventional DC2 (CD11b^+^), and T cells (CD3^+^) from BALB/c splenocytes.

Previously, we demonstrated that a single DNA immunization with APC-targeted HA from H1N1 influenzas provided protection against a lethal challenge in mice (23, 24). A key feature of the vaccine was the bivalent display of antigens that were linked via a dimerization unit containing the hinge region and C_H_3 from human IgG3 to targeting units specific for receptors on APCs (Fig. 1A) (28, 32). Here, we inserted HA from A/chicken/Italy/13474/99 (H7N1) (amino acids 19 to 536 [aa19-563]) into the same vaccine format and targeted HA toward either chemokine receptors 1, 3, and 5 (CCR1/3/5), or MHC-II molecules (Fig. 1A). To target HA to MHC-II molecules, we used a single-chain-variable fragment (scFv) that was specific for I-E^d^ (denoted αMHCII-H7), whereas the chemokine Mip1α was used for targeting of HA to CCR1/3/5 (denoted Mip1α-H7). As nontargeted controls, we constructed a vaccine where the APC-specific targeting unit was replaced with a scFv against the hapten Nip (denoted αNip-H7) and a plasmid encoding only HA (denoted H7). In some experiments, we also used the previously described MHC-II-targeted vaccine with HA from influenza A/Puerto Rico/8/34 (H1N1) (24) to control for antigen specificity (denoted αMHCII-H1).

Supernatants from 293E cells transiently transfected with the different vaccine plasmids confirmed the appropriate size (Fig. 1B) and *in vitro* expression of the secreted vaccine proteins (Fig. 1C). The nontargeted control, αNip-H7, exhibited an ∼2-fold-higher protein expression level than the other vaccines, potentially providing a concentration dependent benefit for the *in vivo* DNA vaccinations. In order to verify efficient binding of the vaccine proteins to APC, we assayed their binding profiles to splenocytes (Fig. 1D). Vaccines targeting MHC-II molecules ligated B cells, macrophages, and conventional DC1 and DC2, whereas CCR1/3/5-targeted vaccines ligated macrophages and conventional DC1. As expected, nontargeted control failed to ligate any of the cell types investigated, and T cells were not bound by either of the constructs.

### An intact multibasic cleavage site increases antibody responses.

**FIG 2:**
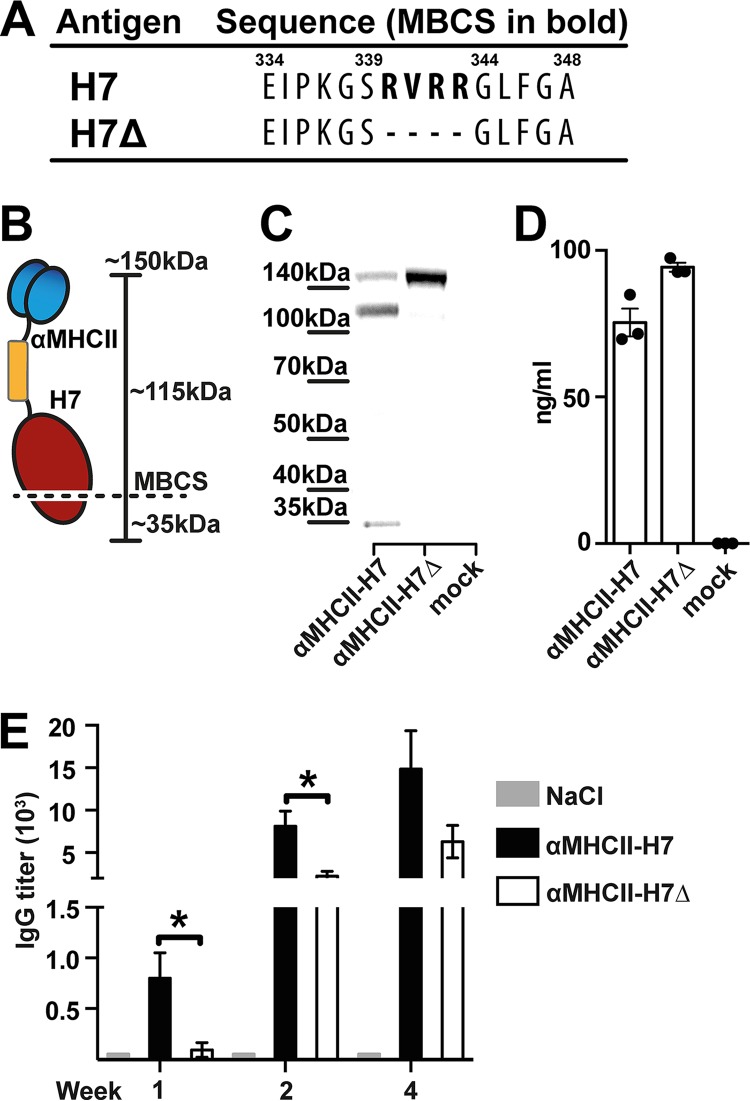
MHC-II-targeted HA vaccination with or without a multibasic cleavage site. (A) Alignment of the MBCS in H7 and the deleted corresponding sequence in H7Δ. (B) Schematic illustration depicting the vaccine monomer with indicated MBCS in HA. The full-length vaccine monomer is ∼150 kDa and HA2 is ∼35 kDa, resulting in fragments of ∼115 and ∼35 kDa, respectively, under reducing conditions. (C) Western blot of secreted vaccine proteins (indicated) under reducing conditions in supernatant from transiently transfected 293E cells. (D) Binding of secreted αMHCII-H7 or αMHCII-H7Δ proteins in supernatants from transiently transfected 293E cells detected in an ELISA specific for HA from A/Shanghai/1/2013 (H7N9). (E) Mice (*n* = 6/group) were vaccinated i.d. with plasmid DNA encoding either αMHCII-H7 or αMHCII-H7Δ, or NaCl, and IgG in sera measured in ELISA against recombinant HA from influenza A/Shanghai/1/2013(H7N9) at weeks 1, 2, and 4 postvaccination. *, *P* < 0.05 (two-tailed Mann-Whitney test).

Conventional production of vaccines against HPAIV depends on removal of the MBCS in order to prevent disease and potential lethality embryonated hens' eggs (4, 33). In contrast, this is not a problem for the synthetic production of DNA vaccines. Therefore, we wanted to compare the immunogenicities of APC-targeted H7 antigens with the intact or deleted MBCS sequences, and we cloned an HA with a deleted MBCS into the MHC-II-targeted vaccine format described above. The vaccine displaying the deleted MBCS sequence was denoted αMHCII-H7Δ (Fig. 2A).

The correct size and expression of αMHCII-H7Δ was confirmed (Fig. 2C and D). Since endogenous proteases in 293E cells will cleave H7 with intact MBCS (22), we observed bands corresponding to cleavage of HA0 into HA1 (∼115 kDa, including the targeting domain and dimerization unit) and HA2 (∼35 kDa) for αMHCII-H7, but not for αMHCII-H7Δ (Fig. 2B and C). The *in vitro* expression levels of αMHCII-H7Δ were slightly higher than that of αMHCII-H7 (Fig. 2D). Nevertheless, *in vivo* DNA vaccination of mice showed that αMHCII-H7 induced significantly higher IgG titers than did αMHCII-H7Δ (Fig. 2E). The difference was significant already 1 week postvaccination, and antibody levels remained higher at least 4 weeks postvaccination. Taken together, the data suggest that H7 with an intact MBCS could offer an immune-potentiating effect that is maintained when targeting of HA to MHC-II molecules in mice. Thus, further experiments were performed with αMHCII-H7.

### Rapidly increased IgG levels after MHC-II-targeted vaccination.

**FIG 3:**
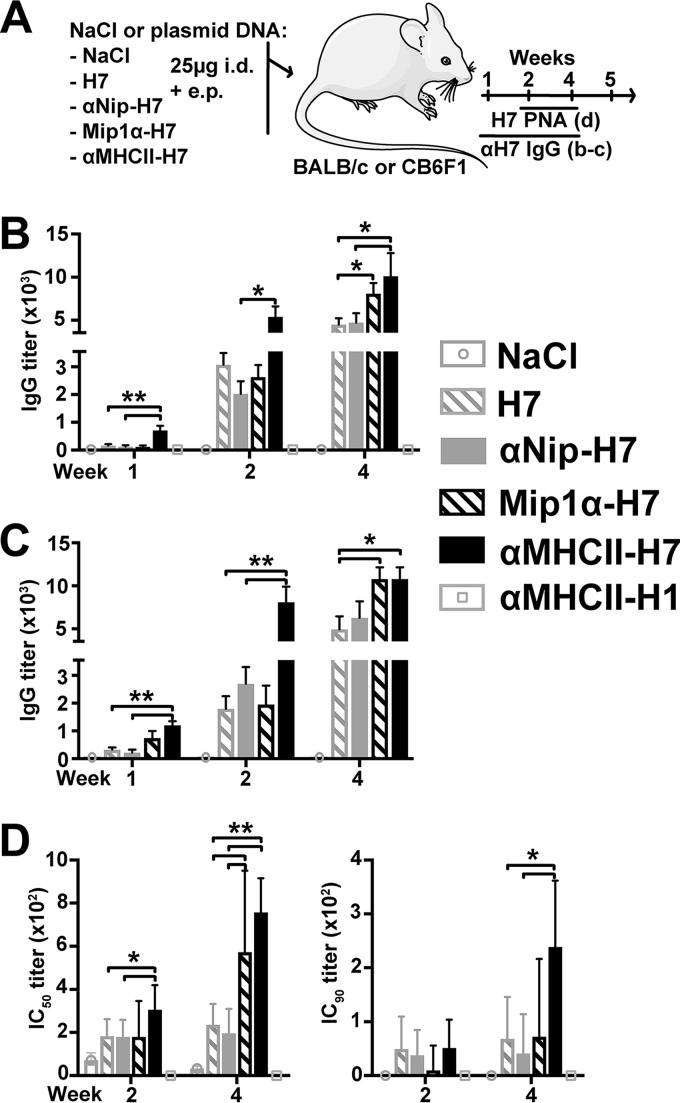
Antibody responses after a single DNA vaccination. (A) Schematic illustration of the experiment. Briefly, BALB/c (*n* = 12/group) or CB6F1 mice (*n* = 6/group) were DNA vaccinated once i.d. with the indicated vaccine plasmids. Sera were collected up to 4 weeks postvaccination from BALB/c (B) and CB6F1 (C), and antibody responses were measured by ELISA against recombinant HA from influenza A/Shanghai/1/2013 (H7N9). *, *P* < 0.05; **, *P* < 0.01 (two-tailed Mann-Whitney test). (D) Sera from BALB/c from weeks 2 and 4 were pooled by group and assayed in a pseudotype microneutralization assay against A/FPV/Rostock/1934 (H7N1). Neutralization curves were fitted with GraphPad Prism 6 software, and the IC_50_ and IC_90_ titers were calculated. *, *P* < 0.05; **, *P* < 0.01 (extra sum-of-squares F test).

Since the rapid induction of protection is crucial for pandemic prevention, mice were DNA vaccinated only once, and antibody responses in serum were assessed for 5 weeks postvaccination. Mice were immunized with plasmids targeting H7 toward either CCR1/3/5 or MHC-II molecules or with nontargeted control vaccines (αNip-H7 and H7). In addition, a group was vaccinated with αMHCII-H1 to control for specificity (Fig. 3A). To generalize the effect of APC targeting, parallel experiments were performed in BALB/c mice and the CB6F1 hybrid strain. Results demonstrated that IgG responses were significantly elevated already the first weeks after vaccination with αMHCII-H7 in both BALB/c (Fig. 3B) and CB6F1 mice (Fig. 3C). The antibody responses after vaccination with Mip1α-H7 were only significantly increased above that of the nontargeted controls at week 4 postvaccination for both mouse strains. As expected, αMHCII-PR8 did not induce any antibodies that cross-reacted with influenza H7. The same trends were observed when the vaccines were delivered intramuscularly (i.m.) in BALB/c (data not shown).

In order to assess the neutralizing antibody response, sera from weeks 2 and 4 postvaccination in BALB/c were examined in a pseudotype-based neutralization assay against A/FPV/Rostock/1934(H7N1) HA and neuraminidase (NA) pseudotype virus. The results demonstrated that a single vaccination with αMHCII-H7 significantly increased 50% inhibitory concentration (IC_50_) levels in sera at both 2 and 4 weeks postvaccination compared to the nontargeted control vaccines (αNIP-H7 and H7) (Fig. 3D). Furthermore, significantly enhanced IC_50_ levels were observed in sera collected 4 weeks after vaccination with Mip1α-H7, compared to the nontargeted controls. The results for both αMHCII-H7 and Mip1α-H7 corresponded to the increases that were observed in enzyme-linked immunosorbent assay (ELISA; *P* < 0.05, Spearman correlation IC_50_ and serum titers) (Fig. 3B and D). When extending the analyses to examinations of IC_90_ titers, vaccination with αMHCII-H7 significantly increased the levels of protective antibodies compared to nontargeted controls. Taken together, the data demonstrated that αMHCII-H7 is superior at induction of neutralizing antibodies compared to Mip1α-H7 and nontargeted controls.

### Vaccine induced production of IFN-γ-secreting cells and cytotoxic T lymphocytes.

**FIG 4:**
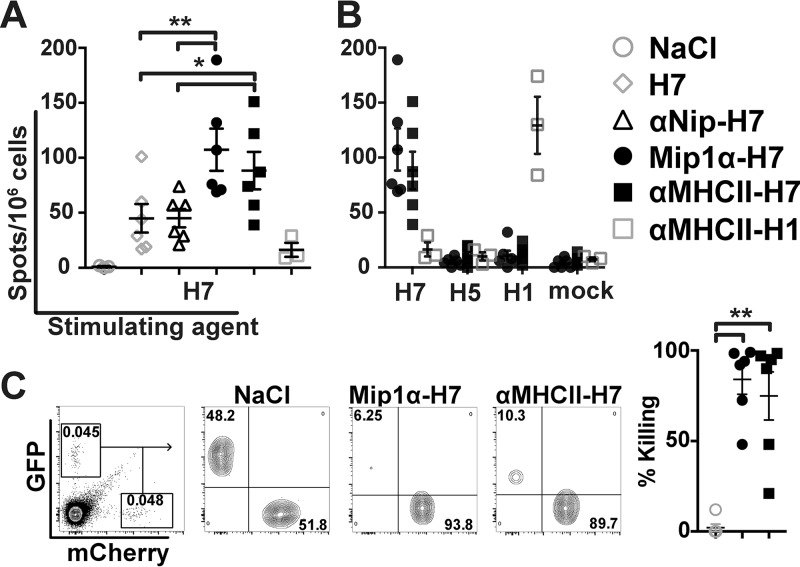
Induction of T cells after a single immunization. BALB/c mice (*n* = 6/group; *n* = 3/group for NaCl and H1 controls) were DNA vaccinated i.m. with plasmids encoding the indicated vaccines. Spleens were harvested at 8 weeks postvaccination. Splenocytes were restimulated with recombinant HA from A/Shanghai/1/2013(H7N9) (A) or HA from either A/Shanghai/1/2013(H7N9), A/Vietnam/1194/2004(H5N1), or A/Puerto Rico/8/34(H1N1) (B), and the numbers of IFN-γ-secreting cells were evaluated. (C) BALB/c mice (*n* = 6/group) were vaccinated i.d. with plasmid DNA encoding the indicated vaccines. After 5 weeks, 5 × 10^6^ A20 cells expressing cytosolic H7 and GFP and 5 × 10^6^ A20 cells expressing cytosolic mCherry were injected i.v. The prevalences of GFP- or mCherry-positive cells were assessed 16 h later, and the killing ratios were calculated. *, *P* < 0.05; **, *P* < 0.01 (two-tailed Mann-Whitney test).

Although antibodies may block an influenza infection, T cells can clear already infected cells. Thus, the different DNA vaccines were assessed for their ability to induce sustained IFN-γ-secreting cells and cytotoxic T lymphocytes (CTL) after immunization. Splenocytes were harvested 8 weeks postvaccination and restimulated with recombinant HA from H7N9 influenza. The results demonstrated that mice receiving either αMHCII-H7 or Mip1α-H7 induced significantly higher levels of IFN-γ-secreting cells compared to the nontargeted controls (Fig. 4A). The enhanced induction was specific for H7, since restimulation with HA from other influenza A subtypes failed to increase IFN-γ production. As an additional control, αMHCII-H1 increased the levels of IFN-γ-secreting cells only after stimulation with HA from H1N1 influenza (Fig. 4B).

To further address whether the increase in IFN-γ-secreting cells could be interpreted as increased CTL activity *in vivo*, we retrovirally transduced an A20 mouse B lymphoma cell line to express cytosolic H7 and GFP, or mCherry as a control. At week 5 postvaccination, the cell lines were injected into BALB/c mice, and the ratio between green fluorescent protein (GFP)- and mCherry-positive cells was investigated 16 h later by flow cytometry. The killing ratios were calculated by comparing the remaining GFP population to the control population expressing mCherry (Fig. 4C). Both APC-targeted vaccines induced significant CTL responses, and in correspondence with the enzyme-linked immunospot assay (ELISPOT) data, CCR1/3/5-targeting induced a higher T-cell response compared to the MHC-II-targeted vaccine. In sum, the data demonstrated that targeting of HA to APCs significantly increased the vaccine induced T-cell responses and that they can participate in protection against an influenza infection.

### A single APC-targeted DNA vaccination can protect mice from a lethal influenza challenge.

**FIG 5:**
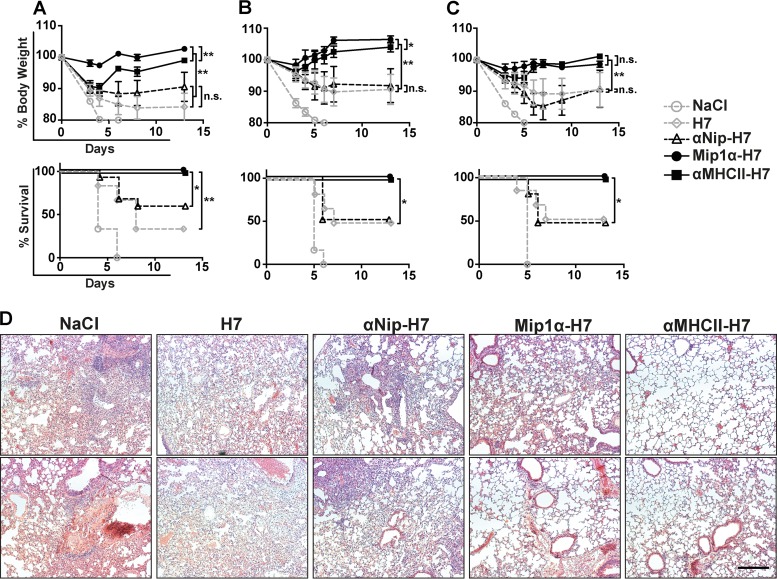
Viral challenge of DNA-immunized mice. BALB/c mice (*n* = 6 to 12/group) or CB6F1 mice (*n* = 6/group) were vaccinated with 25 μg of DNA i.d. and challenged with 20 × LD_50_ of mouse-adapted A/turkey/Italy/3889/1999 (H7N1) at week 5 postvaccination. The body weight (upper panels) was measured after challenge to assess morbidity, and survival curves (lower panels) are shown for mice receiving the indicated vaccines from BALB/c mice (A) and CB6F1 mice (B). (C) BALB/c mice were vaccinated with vaccines encoding the HA antigen A/turkey/Italy/3889/1999 (H7N1) homologous to the challenge strain and challenged at week 5 postvaccination. Weight curves: *, *P* < 0.05; **, *P* < 0.01 (two-way ANOVA). Survival curves: *, *P* < 0.05; **, *P* < 0.01 (Gehan-Breslow-Wilcoxon test). (D) Representative micrographs of H&E-stained sections of lungs from each group from the experiment in panel A collected 7 days postchallenge. Scale bar, 250 μm.

In order to examine whether the APC-targeted vaccines could rapidly confer protection against influenza, mice were DNA immunized once intradermally (i.d.) and then challenged with a lethal dose of influenza A/turkey/Italy/3889/1999 (H7N1) 5 weeks after vaccination. Mice that received the APC-targeted vaccines showed significantly less morbidity compared to the nontargeted controls, and both BALB/c (Fig. 5A), and CB6F1 mice (Fig. 5B) were fully protected against influenza. In contrast, mice that received saline or nontargeted controls rapidly lost weight, and 40 to 60% of the mice receiving the nontargeted control vaccines had to be euthanized within 8 days postchallenge.

The influenza strain used for challenge has some sequence differences compared to the vaccine strain. In order to examine protection in a homologous challenge model, we also immunized mice with HA from A/turkey/Italy/3889/1999 (H7N1) and challenged them with this strain 5 weeks later (Fig. 5C). Mice receiving APC-targeted vaccines were fully protected against the lethal influenza challenge, in contrast to the nontargeted control vaccines. For the heterologous challenge experiments above (Fig. 5A and B), APC targeting of HA significantly improved protection against the influenza challenge compared to the nontargeted control vaccines, but we also observed an increased weight loss in mice receiving MHC-II-targeted HA compared to the group vaccinated with Mip1a-H7. Here, we observed no significant difference in weight loss after MHC-II- and CCR1/3/5-targeted vaccination in the homologous challenge (Fig. 5C).

In order to further examine the differences in protection after vaccination with MHC-II- and CCR1/3/5-targeted HA, we set up an experiment examining lung pathology after viral challenge in BALB/c mice. Thus, mice were immunized with HA from A/chicken/Italy/13474/1999 (H7N1) and challenged 5 weeks later with A/turkey/Italy/3889/1999 (H7N1). At day 7 postchallenge, lungs were harvested, sectioned and stained with hematoxylin and eosin (H&E). Lungs harvested from control mice receiving NaCl and nontargeted control vaccines displayed histiocytic alveolitis, interstitial pneumonia, and edema. In contrast, mice receiving APC-targeted vaccines had healthy lungs with only minor lung pathology (Fig. 5D). Further, we observed that lungs collected from mice vaccinated with Mip1α-H7 showed more cellular infiltration and lung pathology compared to mice vaccinated with αMHCII-H7.

### Targeted vaccines induce T-cell responses that contribute to protection against viral challenge.

**FIG 6:**
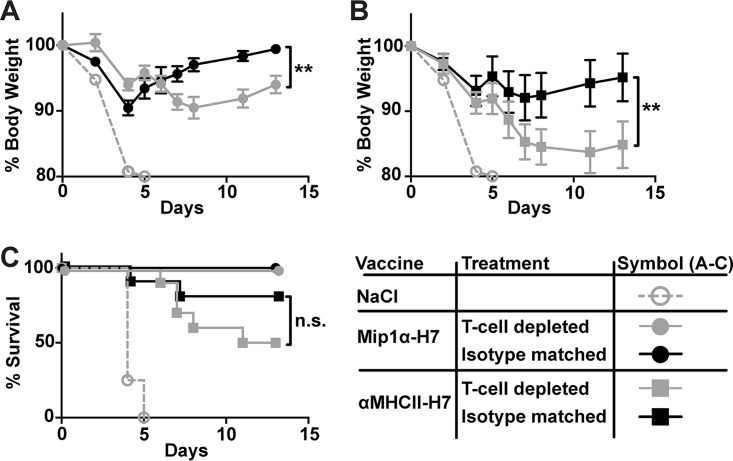
Viral challenge of DNA-immunized mice after depletion of T cells. Mice (*n* = 8 to 10/group) were DNA vaccinated i.d. with the indicated vaccines and then challenged with 20 × LD_50_ A/turkey/Italy/3889/1999 (H7N1) 5 weeks postvaccination. Two days prior to challenge—and every other day until completion of the experiment—mice were injected i.p. with either CD4 and CD8 depleting MAbs or isotype-matched MAbs. Starting at the day of challenge (D0), weight was monitored in mice vaccinated with Mip1α-H7 (A) or αMHCII-H7 (B). *, *P* < 0.05; **, *P* < 0.01 (two-way ANOVA). (C) Survival curves for mice in panels A and B. *, *P* < 0.05; **, *P* < 0.01 (Gehan-Breslow-Wilcoxon test).

In order to investigate whether the main mediator of protection was antibodies or T cells, immunized mice were depleted of CD4^+^ and CD8^+^ T cells just prior to a lethal influenza challenge (Fig. 6). The depletion was maintained by injections of CD4- and CD8-depleting antibodies every other day and was determined to have an efficacy level of >99% (data not shown). T-cell-depleted mice showed no significant increase in mortality compared to mice receiving isotype matched control antibodies. This demonstrated that the induced antibodies were protective. However, recovery after infection was significantly impaired in T-cell-depleted mice, as shown by the significant differences in morbidity that were observed during the second week after infection. The experiment highlights the importance of T cells in clearing a heterologous influenza infection. In conclusion, the APC-targeted vaccination induced both antibodies and T cells that contribute to protection against influenza.

## DISCUSSION

In the event of a pandemic outbreak of HPAIV, a vaccine platform capable of launching a quick production and response is crucial. It is essential to rapidly induce protective immunity in the population to limit viral spread and disease. We have here demonstrated that DNA vaccines targeting HA from HPAIV H7 to APCs in mice represent a potential vaccine candidate for induction of rapid immunity. Importantly, the APC-targeted DNA vaccines could mediate protection in mice that were challenged with a lethal dose of influenza H7N1 virus only 5 weeks after vaccination.

We have here used HA from either A/turkey/Italy/3889/1999 (H7N1) or A/chicken/Italy/13474/1999 (H7N1) for vaccination, and we observed that both vaccines can confer protection against a viral challenge with A/turkey/Italy/3889/1999 (H7N1). Thus, the induced immune responses can to some extent confer heterologous protection against different H7 strains. Although this cannot at present be generalized to more H7 strains, we also found that vaccination with HA from A/chicken/Italy/13474/1999 (H7N1) raised neutralizing antibodies against A/FPV/Rostock/1934 (H7N1) in a pseudotype-based neutralization assay.

In order to further investigate the mechanisms behind protection, mice vaccinated with Mip1α-H7 or αMHCII-H7 were injected with depleting antibodies against CD4^+^ and CD8^+^ T cells or isotype-matched control antibodies. After challenge with the H7N1 virus, no significant differences were observed in survival between the T-cell-depleted mice and the isotype-treated mice. Although this indicated that cross-reactive antibodies contributed to the protection, T-cell-depleted mice showed a significant increase in morbidity and delayed recovery postchallenge. Presumably, the vaccine induced neutralizing antibodies that could recognize the heterologous virus were below the limiting threshold necessary to mediate complete sterile protection at the given dose. In this situation, a recall of a neutralizing memory B-cell repertoire during the first days of infection likely inhibited ongoing viral infections, but the lack of viral clearance in already infected cells led to some disease manifestations. This observation held true both for mice vaccinated with αMHCII-H7 and for mice vaccinated with Mip1α-H7.

Previously, we have demonstrated that targeting of antigen to MHC-II molecules is particularly efficient for induction of antibodies, whereas targeting of antigen to CCR1/3/5 more efficiently will induce T-cell responses (25). After vaccination with A/chicken/Italy/13474/1999 (H7N1) and challenge with A/turkey/Italy/3889/1999 (H7N1), we observed that mice vaccinated with Mip1α-H7 had a significantly reduced weight loss compared to αMHCII-H7. In contrast, we found no significant difference in weight loss between mice vaccinated with αMHCII-H7 or Mip1α-H7 when HA from the homologous strain was used. T cells have an increased potential for mediating cross-protection within variations of antigenic drift (34), since T-cell epitopes are more conserved across different subtypes (35). It is therefore likely that increased numbers of IFN-γ-secreting T cells and the slight increased CTL response after Mip1α-H7 vaccination, compared to αMHCII-H7, can account for the difference in weight loss observed after heterologous challenge. Furthermore, the antibody response after Mip1α-targeted vaccine delivery is dominated by IgG2a (25). Nonneutralizing H7-specific IgG2a antibodies have also been demonstrated to be protective in mice (36, 37). Antigen-specific antibodies of the IgG2a isotype efficiently induce Fc-receptor-mediated effector functions such as antibody-dependent cell-mediated cytotoxicity (ADCC) (38, 39), complement-dependent cytotoxicity (40, 41), or antibody-dependent cell phagocytosis (42, 43). In addition, ADCC triggering antibodies have been shown to be cross-reactive (44) and might be of particular importance in a pandemic setting (44, 45). In summary, both MHC-II and CCR1/3/5 targeting vaccines induced humoral and cellular responses that contributed to the mode of the observed protection.

Targeting of H7 to APCs consistently increased immune responses, compared to nontargeted controls. Of note, a direct ligation between antigen and targeting unit has been found a prerequisite for efficient induction of immunity (46). It is possible that binding of an APC-targeted vaccine to an APC will improve access to antigenic epitopes and as such promote more efficient binding to B-cell receptors. A stronger and more specific ligation of DCs or B cells might drive an accelerated formation of germinal centers with subsequent isotype switching and affinity maturation. Furthermore, the APC-targeted vaccine could bridge APCs and B cells in a synapse that promotes efficient and mutual activation (47, 48). Furthermore, a synapse formation between an APC and a B cell could lead to B cells sampling antigen from the top, i.e., the head region of HA. This might lead to selection B cells recognizing more neutralizing epitopes in HA resulting in an antibody repertoire with highly neutralizing capacity, as observed with the MHC-II-targeted vaccine.

Our data have clearly demonstrated the benefit of targeting antigen to APCs, particularly when the aim is to induce rapid immune responses to limit the outbreak of pandemic influenza. Previous studies have demonstrated that the immune potentiating effect when targeting to APCs is particularly prominent for weak antigens (28, 32). In contrast, HA from HPAIV H7 is immunogenic in mice, and vaccination with the H7 antigen alone was able to induce modest IgG titers over time. It is possible that avian HAs have increased immunogenicity compared to human HAs due to differences in glycosylation patterns that would stimulate production of immunostimulatory agents in DCs (49). In addition, inactivated influenza viruses with an α2,3 preference for sialic acid receptors have been shown to induce higher levels of proinflammatory cytokines in human DCs (18). Thus, HA from HPAIV might trigger an innate immune response that translates into activation of adaptive immunity in mice. However, the antibody levels observed after immunization with nontargeted controls developed significantly more slowly than for the APC-targeted vaccines. Furthermore, the nontargeted controls do not enhance immune responses to the same extent as the APC-targeted vaccines and, most importantly, only APC-targeted vaccines could induce protective immunity, after a single vaccination, toward a lethal challenge with influenza virus.

When using conventional vaccine manufacture for selected avian influenza strains, removal of the MBCS and exchange of certain internal genes is required (4). However, removal of the MBCS can render the virus low pathogenic and poorly immunogenic, compromising vaccine efficacy with obvious implications for vaccine design against pandemic outbreaks (17, 50). Here, we also found that deletion of the MBCS was associated with reduced immunogenicity, which again highlights a benefit of using DNA-based vaccines. Ubiquitous proteases will cleave H7 in the vaccine molecules *in vivo*. Cleavage of HA in the APC-targeted vaccine molecule results in efficient presentation of the native head region in HA1. Vaccines expressed with uncleaved HA0 might lack some of the essential structural epitopes in HA1 or the HA1-HA2 interface. The majority of protective antibodies bind to the globular head domain of HA. Thus, the cleaved H7 format could more efficiently present epitopes from native HA1, potentially resulting in a more stringent selection of B cells with neutralizing capacity. This effect is probably tissue and species dependent (23, 51), but it is a general benefit from DNA vaccines.

We have here demonstrated that a single DNA vaccination with APC-targeted H7 can confer protection against H7 influenza in mice. No other vaccine format can at present compete with the speed of production and deployment that is possible for DNA vaccines. For prophylactic mass vaccinations, DNA vaccines can be delivered without adjuvant to the dermis by noninvasive needle-free jet delivery systems (26, 52). Ideally, the DNA vaccine should be tailored to precisely match an emerging pandemic strain, which is also a feasible strategy given the short time in which DNA vaccines can be mass produced. The rapid induction of protective antibodies observed when targeting H7 to MHC-II molecules highlights this receptor as particularly interesting for construction of pandemic DNA vaccines. For broader protection, it could be beneficial to target the H7 to chemokine receptors. In summary, we have here demonstrated that DNA delivery of an APC-targeted vaccine could greatly aid the control of an unexpected pandemic threat.

## MATERIALS AND METHODS

### Molecular cloning.

The HA genes from A/chicken/Italy/13474/1999 (H7N1) (aa19-536) and A/turkey/Italy/3889/1999 (H7N1) (aa19-536) were ordered with flanking SfiI sites (GenScript, Piscataway, NJ) and cloned into the previously described hCMV-based pLNOH_2_ vaccine vectors equipped with targeting units consisting of either a single-chain-variable fragment (scFv) specific for MHC-II (I-E^d^) (32), an scFv specific for the hapten 4-hydroxy-3-iodo-5-nitrophenylacetic acid (NIP) (nontargeted control) (32), or the macrophage inflammatory protein 1α (Mip-1α) (25, 28). In another vaccine construct, the multibasic cleavage site (aa340-343) was removed from HA (H7Δ), and the gene was cloned into a vaccine vector with an MHC-II-specific scFv. In addition, a vaccine construct encoding only HA was constructed by introduction of an upstream BsmI restriction site with primers the 5′-GGTGTGCATTCCGGCCTCGGTG and 3′-GTGGATCCTCTAGAGTCGACGGACCGGC (the BsmI site and the start of the SfiI site are underlined).

### Assessment of *in vitro* expressed vaccine proteins.

An influenza A H7N9 (A/Shanghai/1/2013) hemagglutinin ELISA pair set (SEK40104; Sino Biological, Inc., North Wales, PA) was used. Briefly, ELISA plates (Costar 3590; Corning, Corning, NY) were coated with 0.5 μg/ml rabbit anti-HA monoclonal antibody (MAb), blocked with 1% bovine serum albumin (BSA), and incubated with supernatants from 293E cells transfected with 1-μg vaccine plasmids and 2 μg of polyethylenimine (PEI). Vaccine proteins were detected with horseradish peroxidase-conjugated anti-HA pAb (1 μg/ml) and 3,3′,5,5′-tetramethylbenzidine (TMB) substrate (CL07; Merck Millipore, San Diego, CA). The reaction was stopped after 20 min of incubation with an equal volume of 0.5 M H_2_SO_4_, and the plates were read at 450 nm with a Tecan reader (Tecan, Switzerland) using Magellan v5.03 software.

Vaccine proteins were produced by transient transfection of 293E cells with PEI. Constructs containing the C_H_3 domain were purified on a CaptureSelect FcXL affinity column (catalog no. 194328005; Life Technologies, Naarden, The Netherlands). Splenocytes were harvested from BALB/c mice, and single cell suspensions were FcγR blocked by incubation with 30% heat-inactivated rat serum and stained with purified vaccine proteins, CD3-VF450 (catalog no. 75-0032; Tonbo Biosciences, San Diego, CA), CD19-FITC (catalog no. 35-0193; Tonbo Biosciences), CD11b-PerCP/Cy5.5 (catalog no. 65-0112; Tonbo Biosciences), CD11c-PE/Cy7 (catalog no. 117318; BioLegend, San Diego, CA), F4/80-AF700 (catalog no. 123130; BioLegend), and CD64-APC (catalog no. 139306; BioLegend), followed by hCH3-PE (catalog no. 409304, BioLegend). Cells were analyzed on an Attune NxT flow cytometer (Thermo Fisher Scientific, Waltham, MA) and FlowJo software.

### Western blotting.

Vaccine plasmids were transiently transfected into 293E cells as described above. Supernatants were up-concentrated (VivaSpin 500, MWCO 10000; Sartorius, Gottingen, Germany) and denatured in sodium dodecyl sulfate sample buffer at 95°C for 5 min. The samples were then run on a Bolt 4 to 12% Bis-Tris Plus gel (NW04122BOX; Novex, Carlsbad, CA), blotted onto a polyvinylidene difluoride (PVDF) membrane (catalog no. IB24001, iBlot transfer stack; Invitrogen, Kiryat Shmona, Israel), blocked in 2% skimmed milk, and detected by using monoclonal (Fig. 1) or polyclonal (Fig. 2) rabbit αH7 antibodies (SEK40104; Sino Biological). Next, the membrane was incubated with polyclonal goat anti-rabbit IgG conjugated to alkaline phosphatase (ALP; A3687; Sigma-Aldrich) and developed with the BCIP/NBT-Purple Liquid substrate system for membranes (B3679; Sigma-Aldrich).

### Mice and cell lines.

Cell work was performed with human embryonic kidney 293E cells purchased from the American Type Culture Collection (ATCC; Manassas, VA, USA). Six- to eight-week-old female BALB/c or CB6F1 mice (Janvier, le Genest-Saint-Isle, France) were used in all experiments. The animals were housed under minimal disease conditions, and all animal experiments were preapproved by the Norwegian Animal Research Authority.

### Vaccination.

Mice were anesthetized (0.1 mg/10 g [body weight] with a cocktail composed of Zoletil Forte [250 mg/ml; Virbac France], Rompun [20 mg/ml; Bayer Animal Health GmbH], and fentanyl [50 μg/ml; Actavis, Germany]) by intraperitoneal (i.p.) injection. For i.d. delivery of vaccines, the lower back region of each mouse was shaved, and 12.5 μg of plasmid in a 25-μl volume was injected at two sites (total DNA/mouse, 25 μg), immediately followed by skin electroporation (DermaVax; Cellectis, Paris, France). For i.m. delivery of vaccines, mice were shaved on each leg, and 6.25 μg of DNA was injected in a 50-μl volume into each quadriceps femoris (total DNA/mouse, 12.5 μg). Immediately after injection, electrical pulses were applied at the injection site (Elgen; Inovio Biomedical Co., Blue Bell, PA). All DNA vaccines were purified by using an EndoFree Plasmid Mega kit (catalog no. 12381; Qiagen, Hilden, Germany) and dissolved in sterile injection fluid (0.9% NaCl).

### Serum ELISA.

Blood was harvested by puncture of the saphenous vein, and sera were isolated by centrifugation. ELISA plates (Costar 3590) were coated with 0.5 μg/ml recombinant HA [A/Shanghai/1/2013 (H7N9)] (40104-V08B; Sino Biological), blocked with 1% BSA, and incubated with serially diluted serum samples assayed individually (*n* = 6 to 12/group). HA-specific antibodies were detected with alkaline phosphatase conjugated goat anti-mouse IgG (A1418; Sigma-Aldrich), developed with phosphatase substrate (P4744; Sigma-Aldrich) and analyzed as previously described. For all serum ELISAs, titers were determined as the last serum dilution with an optical density at 405 nm above background (the mean absorbance from NaCl-vaccinated mice added five times the standard error of the mean for the group).

### Pseudotype-based neutralizing assay.

Pseudotyped virus was prepared and quantified as previously reported (53). Briefly, 3 × 10^3^ MDCK cells were seeded in each well of a 96-well culture plate (Corning) and incubated overnight at 37°C in 5% CO_2_ and saturated humidity. Then, serially diluted serum samples, pooled by group, that had been preincubated with HA and NA pseudotypes from A/FPV/Rostock/1934 (H7N1) (2,000 to 200,000 relative luciferase activity [RLA]) at 37°C in 5% CO_2_ and saturated humidity for 1 h were added to the cells for 72 h of incubation at 37°C in 5% CO_2_ and saturated humidity. The RLA values were measured by a BrightGlo luciferase assay according to the manufacturer's instructions (Promega, Madison, WI). The percent inhibition was calculated as follows: (RLA in pseudotypes and medium control − RLA in pseudotypes and immune serum at a given dilution)/RLA in pseudotypes and medium control. The data were fitted to a sigmoidal dose-response curve using GraphPad Prism 6 software, and IC_50_ and IC_90_ values were determined from those data sets.

### ELISPOT assay.

Mouse IFN-γ ELISPOT Plus plates (3321-4APT; Mabtech, Nacka Strand, Sweden) were blocked with RPMI plus 10% fetal calf serum for 2 h at 37°C in 5% CO_2_. Mouse spleens were harvested at 8 weeks postinfection, and single cell suspensions were prepared with a gentleMACS dissociator (Milteny Biotec, Bergisch Gladbach, Germany), followed by incubation in ACT lysis buffer for 5 min on ice. Next, 0.5 × 10^6^ cells were seeded per well, and cells were stimulated with either medium (negative control), ConA (1 μg/ml, positive control), or 10 μg/ml recombinant HA from either A/Shanghai/1/2013 (H7N9), or A/Puerto Rico/8/1934 (H1N1), or A/Vietnam/1194/2004 (H5N1) (40104-V08B, 11684-V08H, and 11062-V08H1, respectively; Sino Biological). Plates were incubated for 20 h at 37°C in 5% CO_2_ before incubation with the detection antibody (3321-4APT; Mabtech) and development with the BCIP/NBT-Purple liquid substrate system for membranes (B3679; Sigma-Aldrich). Plates were analyzed with the CTL-ImmunoSpot analyzer (CTL, Shaker Heights, OH).

### *In vivo* killing assay.

A20 cells expressing cytosolic H7 and GFP, as well as control cells expressing mCherry, were created by retroviral transduction, followed by selection of highly expressing cells by fluorescence-activated cell sorting. H7 GFP cells and mCherry cells (5 × 10^6^; 1:1) were injected intravenously (i.v.) into BALB/c mice, and the prevalence of GFP-positive and mCherry-positive cells in the spleen were analyzed 16 h later in an Attune NxT flow cytometer (Thermo Fisher Scientific, Waltham, MA) using FlowJo software. Killing ratios were calculated by defining the average ratio between the two cell lines in the NaCl group as 0% killing and finding no H7 GFP cells as 100% killing.

### Viral challenge.

Mice were anesthetized as described previously and inoculated with 20 × LD_50_ mouse-adapted A/turkey/Italy/3889/1999 (H7N1) in 10 μl/nostril. Mice were monitored for weight loss, and mice that lost >20% of the original body weight were euthanized by cervical dislocation.

### H&E staining of lung sections.

Formalin-fixed lungs were embedded in paraffin, sectioned, and stained with H&E. Sections were mounted using Dako toluene-free mounting medium (CS705; Dako, Santa Clara, CA). Micrographs of tissue sections were collected using Nikon Eclipse Ti-S microscope (Nikon Corporation, Tokyo, Japan), and a 10×/0.30 objective lens.

### T-cell depletion.

Mice (*n* = 10/*n* = 8 [NaCl group]) were vaccinated once i.d. as previously described. Two days prior to challenge and then every other day until completion of the experiment, 200 μg of anti-CD4 MAb (GK1.5; ATCC) and 200 μg of anti-CD8 MAb (TIB105; ATCC), or control MAbs (200 μg of SRF8-B6 and 200 μg of Y13-238) (23), were injected i.p. into the mice. At the end of the experiment, the spleens were harvested to assess T-cell depletion. Briefly, single cell suspensions were prepared, and cells stained with fluorescein isothiocyanate-conjugated anti-mouse CD3e (catalog no. 35-0031; Tonbo Biosciences), PerCP-Cy5.5-conjugated rat anti-mouse CD45R (catalog no. 552771; BD Pharmingen), APC-conjugated rat anti-mouse CD4 (catalog no. 1540-11; Southern Biotech Associates, Birmingham, AL), and PE-conjugated rat anti-mouse CD8a (553033; BD Pharmingen). For background assessments, the following isotype controls were used: APC-conjugated rat IgG2b (catalog no. 0118-11; Southern Biotech Associates, Birmingham, AL) and PE-conjugated rat IgG2a (catalog no. 553930; BD Pharmingen). The data were analyzed with FlowJo 10.2 software. Representative flow panels of the stained splenocytes are shown in Fig. S3 in the supplemental material. The degree of depletion was >99%.

### Statistical analysis.

*P* values represent exact values calculated by unpaired nonparametric two-tailed Mann-Whitney tests. Data treated with sigmoidal fitting software are represented with *P* values from the comparison by the extra sum-of-squares F test to determine significance of the inhibitory concentration (IC) levels. Correlation was computed using nonparametric Spearman correlation and represented with a two-tailed *P* value. Weight curves were analyzed with two-way analysis of variance (ANOVA), and survival curves were analyzed with the Gehan-Breslow-Wilcoxon test. All analyses were performed using GraphPad Prism 6 software.
